# Knowledge, attitudes and anxiety towards influenza A/H1N1 vaccination of healthcare workers in Turkey

**DOI:** 10.1186/1471-2334-10-281

**Published:** 2010-09-23

**Authors:** Esen Savas, Derya Tanriverdi

**Affiliations:** 1 Gaziantep University, Faculty of Medicine, Department of Internal medicine, Gaziantep, Turkey; 2 Gaziantep University, Faculty of Health Sciences, Department of Psychiatric Nursing, Gaziantep, Turkey

## Abstract

**Background:**

This study aimed to analyze the factors associated with knowledge and attitudes about influenza A (H1N1) and vaccination, and possible relations of these factors with anxiety among healthcare workers (HCW).

**Methods:**

The study used a cross-sectional descriptive design, and it was carried out between 23 November and 4 December 2009. A total of 300 HCW from two hospitals completed a questionnaire. Data collection tools comprised a questionnaire and the State-Trait Anxiety Inventory (STAI).

**Results:**

Vaccination rate for 2009 pandemic influenza A(H1N1) among HCW was low (12.7%). Most of the respondents believed the vaccine was not safe and protective. Vaccination refusal was mostly related to the vaccine's side effects, disbelief to vaccine's protectiveness, negative news about the vaccine and the perceived negative attitude of the Prime Minister to the vaccine. State anxiety was found to be high in respondents who felt the vaccine was unsafe.

**Conclusions:**

HCW considered the seriousness of the outbreak, their vaccination rate was low. In vaccination campaigns, governments have to aim at providing trust, and media campaigns should be used to reinforce this trust as well. Accurate reporting by the media of the safety and efficacy of influenza vaccines and the importance of vaccines for the public health would likely have a positive influence on vaccine uptake. Uncertain or negative reporting about the vaccine is detrimental to vaccination efforts.

## Background

The earliest confirmed case of influenza A/H1N1 (Swine flu) in 2009 was reported in Mexico in March, and the World Health Organization declared the disease to be a pandemic-Phase 6 on 11 June [1,2].

According to WHO, as of 22 November 2009 the number of deaths resulting from pandemic H1N1 was 7826. The swine flu outbreak also affected Turkey. As of 22 November 2009, the number of deaths due to the virus was 127 in Turkey [1].

Influenza vaccines are one of the most effective ways to protect people from contracting illness during influenza epidemics and pandemics. Other preventive measures are using a mask; washing hands regularly with soap and water; aerating the environment; avoiding hugging, kissing and shaking hands; avoiding touching mouth and nose; avoiding close contact with people [1].

On 13 July 2009, the WHO also recommended that all countries should immunize their HCW as a first priority to protect the essential health infrastructure [2-4]. The potential benefits of influenza vaccination for HCW are three fold -- personal protection, protection of patients, and reduction of absenteeism [5].

Influenza A (2009 H1N1) vaccination of risk groups, primarily HCW, began on 2 November 2009 in Turkey [1]. Meanwhile, "The Prime Minister's personal refusal of getting vaccinated" and "vaccine's unsafety" appeared in Turkish media as negative news [6-12]. It can be considered that this news had an impact on the public and HCW. Negative news on the vaccine can affect people's level of anxiety related to the situation (vaccine). That HCW do not get vaccinated can be an important obstacle to success of campaigns. For this reason, in this study we aimed at researching the reasons why HCW did not to get vaccinated and the possible relation of this situation to anxiety. In Turkey there has been no study addressing the knowledge and attitudes towards influenza A/H1N1 vaccination. Thus, we carried out this study to analyze the factors associated with knowledge and attitudes towards influenza A (H1N1) and anxiety levels among HCW in two hospitals from a south-eastern city of Turkey: Gaziantep.

## Methods

### Participants

The study used a cross-sectional descriptive design and it was carried out between 23 November and 4 December 2009. A structured, self-administered, anonymous questionnaire was disributed to a convenience sample of 300 HCW. Participants comprised doctors (98), nurses (101) and allied health professionals (101) working in two hospitals (University Hospital [UH] and State Hospital [SH]). They entered the study based on their acceptance to the questionnaire. The majority of participants in this study were willing to contribute to this study and only twenty HCW refused to participate. These people refused to participate in the study mainly because of insufficient time.

### Data Collection

The data collection tools comprised a questionnaire on demographic characteristics and the State-Trait Anxiety Inventory (STAI). The completed questionnaires were collected by the researchers (E.S. and D.T.) in the hospitals. According to declarations of the respondents, average time to complete the questionnaire was nearly 10 minutes. All of the participants completed the questionnaire.

#### Survey Items

The questionnaire was composed of questions regarding influenza A/H1N1, the importance of the vaccine, reasons for avoidance of getting vaccinated or acceptance of getting vaccinated.

#### Anxiety

Anxiety was measured by means of the STAI [13]. This self-report questionnaire consists of two subscales each containing 20 items. The state anxiety subscale measures the anxiety at the moment of scoring. State anxiety is conceptualized as a transient emotional condition of the individual, characterized by subjectively experienced feelings of tension, together with a heightened activity of the autonomous nervous system. Trait anxiety measures dispositional anxiety or anxiety in general. Trait anxiety refers to anxiety proneness; that is, relatively stable individual differences in the tendency to react with a more intense state anxiety in situations that are perceived as threatening. The items are summed per scale and transformed into scores between 20 and 80. Higher scores on the STAI indicate a higher intensity of anxiety. The Turkish version of the STAI has been validated previously [14].

### Statistical Analysis

The data were analyzed using SPSS version 13.0. Statistical analyses were used percentage calculation, one-way ANOVA, t-test, and chi-square test. Moreover, multivariate analysis was applied to five variables (Sex, thoughts about the seriousness of the outbreak, protectiveness of the vaccine, safety of the vaccine, whether respondents would allow their children to get vaccinated) that may affect the attitudes of HCW towards vaccination, and the strength of association was expressed as odds ratios with 95% confidence intervals. The level of significance was set at p < 0.05.

### Ethical Considerations

Written permission to conduct this study in hospitals was obtained from the managers. Written information was given to the participants and their oral consent was obtained. The HCW were informed about the purpose of the research and assured of their right to refuse to participate in or to withdraw from the study at any stage. Anonymity and confidentiality of subjects' data were guaranteed.

## Results

The average age of the participants was 31.21, and 53.7% of the sample was women. A total of 60.3% asserted that they considered swine flu as a serious outbreak, and 51.0% agreed that influenza A was a fatal disease. 82.3% stated that they did not find the vaccine protective, and 89.7% expressed a view that the vaccine was not safe, and 86.0% asserted that they did not allow their children to get vaccinated, 44.0% specified that some complications related to the vaccine in their work places occurred.

Among personal measures for protection from swine flu; participants expressed that they took measures like hand washing, avoiding close contact, and using a mask with the rates of 95.7%, 78.7%, 53.3% respectively. On the other hand, as being the most effective preventive measure vaccination was rated only 12.7%. Among vaccinated participants side effects related to the vaccine were considerably low. The most frequently seen side effects were the pain (28.9%), and swelling (7.9) and redness (2.6%) in vaccination site and fewer (18.4%).

Distribution of the answers of the vaccinated and non-vaccinated respondents regarding the knowledge and attitudes about the vaccine and virus are shown in Table 1.

**Table 1:** Distribution of the answers of the vaccinated and non-vaccinated respondents regarding the knowledge and attitudes about the vaccine and virus

	Correct		Total	P*
	**Vaccinated (n = 38)**	**Not vaccinated (n = 262)**		

	**n/%**	**n/%**		

What is the name of the virus?	36(12.9)	243 (87.1)	279	p > 0.05

What are the modes of transmission of virus?	37 (13.6)	236 (86.4)	273	p > 0.05

What is the type of the vaccine?	16 (11.3)	126 (88.7)	142	p > 0.05

	**Yes**		**Total**	P*

	**Vaccinated (n = 38)**	**Not vaccinated (n = 262)**		

Are there any complications related to the vaccine in your workplace or environment ?	14 (10.6)	118 (89.4)	132	p > 0.05

Is swine flu a serious outbreak?	33 (18.2)	148 (81.8)	181	p < 0.001

Is swine flu a fatal disease?	24 (15.7)	129 (84.3)	153	p > 0.05

Is the vaccine protective?	22 (41.5)	31 (58.5)	53	p < 0.001

Is the vaccine safe?	18 (58.1)	13 (41.9)	31	p < 0.001

Will you permit your children to get vaccinated?	20 (47.6)	22 (52.4)	42	p < 0.001

**Personal measures against the disease**				

Using a mask	15 (10.7)	125 (89.3)	140	p > 0.05

Hand washing	1 (7.7)	12 (92.3)	13	p > 0.05

Avoiding indoors and crowded places	17 (12.1)	123 (87.9)	140	p > 0.05

Avoiding close contact	10 (15.6)	54 (84.4)	64	p > 0.05

Others	34 (12.9)	230 (87.1)	264	p > 0.05

**The most common reasons to get vaccinated**	**Yes**			

Being in a risk group	28/73.7			

Declarations of the Ministry of Health	11/28.9			

Death news in media	9/23.7			

Others	6/15.8			

Vaccination of the Ministry of Health	4/10.5			

**The factors influential in refusal to get vaccinated**		**Yes**		

Vaccine's side effects		176/67.2		

Not believing vaccine's protectiveness		146/55.7		

The Prime Minister's refusal of getting vaccinated		81/39.9		

Negative news about vaccine in media		98/37.4		

To be infected by influenza A before		31/11.8		

Others		31/11.8		

*: Chi-Square Test

**Table 2:** Percentage of getting vaccinated and not getting vaccinated according to some variables.

		The state of being vaccinated			
		**Yes *N *(%)**	**No *N *(%)**	*P*-value^*^	**OR (95% CI)**

**Sex**	Women	15(9.3)	146 (90.7)	p > 0.05	0.708[0.471-1.065]
	
	Men	23 (16.5)	116 (83.5)		

**Is swine flu a serious outbreak?**	Yes	33 (18.2)	148 (81.8)	p < 0.001	1.537[1.306-1.810]
	
	No	5 (4.2)	114 (95.8)		

**Is the vaccine protective?**	Yes	22 (41.5)	31 (58.5)	p < 0.001	4.893[3.191-7.503]
	
	No	16 (6.5)	231 (93.5)		

**Is the vaccine safe?**	Yes	18 (58.1)	13 (41.9)	p < 0.001	9.547[5.100-7.871]
	
	No	20 (7.4)	249 (92.6)		

**Will you permit your children to be vaccinated?**	Yes	20 (47.6)	22 (52.4)	p < 0.001	6.268[3.798-0.344]
	
	No	18 (7.0)	240 (93.0)		

OR: Odds Ratio CI: Confidence Interval *: Chi-Square Test

Percentage of getting vaccinated and not getting vaccinated according to some variables are shown in Table 2. Accordingly, the participants who considered that the outbreak was serious, and ones who allowed their children to get vaccinated, and those who believed in the protectiveness and safety of the vaccine got vaccinated more than others. These differences are at a significant level (p < 0.001).

**Table 3:** Comparison of some variables in terms of sex, job title, and work site

	Sex		P^*^	Job Title			P^*^	Work site		P^*^
	**W (n)**	**M (n)**		**Doctor (n)**	**Nurse (n)**	**AHW (n)**		**UH (n)**	**SH (n)**	

What is the name of the virus ? Correct/ Wrong	150/ 11	129/10	p > 0.05	96/ 2	99/ 2	84/ 17	p < 0.001	137/ 13	142/ 8	p > 0.05

What are modes of transmission of virus? Correct/ Wrong	145/ 16	128/ 11	p > 0.05	97/ 1	94/ 7	82/ 19	p < 0.001	141/ 9	132/ 18	p > 0.05

What is the type of vaccine? Correct/ Wrong	74/ 87	84/ 55	p < 0.01	70/ 28	45/ 56	43/ 58	p < 0.001	80/ 70	78/ 72	p > 0.05

Is the vaccine protective? Yes/ No	20/ 141	33/ 106	p < 0.01	35/ 63	11/ 90	7/ 94	p < 0.001	25/ 125	28/ 122	p > 0.05

Is the vaccine safe? Yes/ No	9/ 152	22/ 117	p < 0.005	17/ 81	5/ 96	9/ 92	p < 0.01	15/ 135	16/ 134	p > 0.05

Are there any complications related to the vaccine in your workplace or environment? Present/ Absent	82/ 79	50/ 89	p < 0.01	54/ 44	49/ 52	29/ 72	p < 0.001	93/ 57	39/ 111	p < 0.001

Will you permit your children to get vaccinated? Yes/ No	12/ 149	30/ 109	p < 0.001	25/ 73	5/ 96	12/ 89	p < 0.001	19/ 131	23/ 127	p > 0.05

W: Women, M: Men, UH: University Hospital, SH: State Hospital, AHW: Allied Health Worker

*: Chi-Square Test

Comparison of some variables in terms of sex, job title and work site are shown in Table 3.

In comparison with men, women were more likely to consider the vaccine as not protective (p < 0.01). They believed the vaccine was not safe (p < 0.005) and allowed their children to get vaccinated at a lower rate (p < 0.001) (Table 3). There was not a significant difference between genders in terms of personal measures taken for protection from influenza A (p > 0.05). However, women applied hand washing more frequently than men (p < 0.001).

When HCW were assessed in terms of anxiety levels, state anxiety levels of women were found to be significantly higher in comparison with men (p < 0.005). State anxiety points of those who found the vaccine less safe were also detected as significantly higher than others (p < 0.01) (Table 4).

**Table 4:** Comparison of anxiety levels according to some variables

	State anxiety (Mean ± SD)	P value	Trait anxiety (Mean ± SD)	P value
Sex				
Women	41.4 ± 10.2	P*<0.005	43.9 ± 8.6	P* > 0.05
Men	37.9 ± 10.9		42.2 ± 9.1	

Job Title				
Doctor	38.6 ± 10.6	P** > 0.05	41.7 ± 10.1	P** > 0.05
Nurse	41.5 ± 10.2		43.9 ± 7.2	
Allied Health Worker	39.2 ± 11.0		43.8 ± 9.1	

Work site				
UH	39.8 ± 10.4	P* > 0.05	42.7 ± 8.5	P* > 0.05
SH	39.8 ± 10.9		43.6 ± 9.3	

Is swine flu a serious outbreak?				
Yes	40.2 ± 10.6	P* > 0.05	43.4 ± 8.9	P* > 0.05
No	39.1 ± 10.8		42.7 ± 8.9	

Is swine flu a fatal disease?				
Yes	40.6 ± 10.2	P* > 0.05	43.9 ± 9.2	P* > 0.05
No	39.0 ± 11.1		42.4 ± 8.5	

Is the vaccine protective?				
Yes	37.6 ± 10.8	P* > 0.05	41.6 ± 10.1	P* > 0.05
No	40.3 ± 10.6		43.5 ± 8.6	

Is the vaccine safe?				
Yes	35.1 ± 10.5	P* < 0.01	41.0 ± 9.0	P* > 0.05
No	40.3 ± 10.5		43.4 ± 8.4	

Were you vaccinated?				
Yes	39.0 ± 11.9	P* > 0.05	42.5 ± 7.8	P* > 0.05
No	39.9 ± 10.5		45.2 ± 9.1	

*: t-test **: ANOVA

## Discussion

HCW knew the name of the virus, modes of transmission and vaccine type correctly with the rates of 93.0%, 91.0% and 52.7%, respectively. It can be said that knowledge of HCW in this matter is good.

Most of the participants expressed that they found the vaccine neither protective nor safe. Similarly, in another study in Hong Kong, 61% of the participants believed that the vaccine wasn't protective, and 63% of them believed that the vaccine was unsafe as clinical experiments were not performed. Evidence about safety and efficacy is critical in determining the prevalence of uptake of vaccination [15]. Thus, HCW and the general public have to be convinced about the vaccine's efficacy and safety.

In this study, only 12.7% of HCW got vaccinated. Although a majority of the participants considered the swine flu outbreak as serious, described it as a fatal disease and had a high level of knowledge about the disease, vaccination rate was very low. Similarly, it was determined that the vaccination rates of HCW were very low in Greece, Germany and Italy [16-18].

In terms of order of importance "Being in risk group", "Declarations of the Ministry of Health" and "Death news in the media" became influential in participants' vaccination. Results of the study of Chor et al. (2009) on HCW are parallel to this study and the most common reasons to uptake the vaccine were stated as "protection demand" and "advice of health authorities" [19]. Based on these studies, we can say that health authorities' announcements and publications made through the media and similar ways have a positive effect on vaccination.

Reasons like "side effects of the vaccine" and "not believing in the protectiveness of the vaccine" "The Prime Minister's personal refusal of getting vaccinated" and "negative news about vaccine in the media" are influential factors in HCW' refusal to get vaccinated. The most important obstacles defined in the study of Chor et al. (2009) were "being anxious about the side effects" and "suspicions about the vaccine's safety". In another study, reasons to refuse the vaccine were expressed as anxiety about the vaccine's safety and efficiency [5]. It was determined that there was anxiety about the safety, efficiency and necessity of the vaccine. In the study of Rachiotis et al. (2010), the main reason to refuse the vaccine was fear of side effects, which was stronger in those who received information on the safety of the vaccine mainly from mass media [16]. Also, in our study, state anxiety levels of people who did not rely on the vaccine were found to be significantly higher than those who relied on the vaccine. However, these people's trait anxiety levels were not detected a significant high level. According to the results of this study, it is important to overcome this anxiety and to enable safety in order to convince people to get vaccinated in vaccination campaigns. In our study the Prime Minister's refusal of vaccine had a negative impact, while the advice of health authorities had a positive effect on vaccination. In this case we can say that different attitudes of the Prime Minister and the Ministry of Health affected the vaccination process negatively. Although we do not have certain data on this matter these different attitudes can be said to increase anxiety levels as to this situation. Consistent attitude of the government in vaccination campaigns can contribute a decrease in public anxiety, and an increase in trust.

In professional terms, doctors knew modes of transmission and type of the vaccine correctly at the highest rate, and allied health workers knew these correctly at the lowest rate. Doctors believed in the vaccine's protectiveness and safety more than the others. Also, in comparison with others, doctors gave permission to their children to get vaccinated at a higher level. In this matter, it can be recommended to give importance to inform allied health workers in vaccination campaigns.

In terms of anxiety levels, a significant difference was not detected between those who were vaccinated and those who were not vaccinated.

### Strengths and weaknesses of this study

To our knowledge, this is the first study conducted to assess vaccination rate and factors associated with vaccine acceptance and their relations with anxiety levels of HCW in literature. It provides some new important information on obstacles in vaccination. This study was conducted only in a city center, and had relatively low number of respondents for a social study.

## Conclusions

According to the results of this study, the vaccination rate was very low. Most of the participants believed that the vaccine was not safe. However, it was determined that reasons to refuse were mostly due to the vaccine's side effects, not believing in the vaccine's protectiveness and the Prime Minister's attitude against the vaccine and negative news about vaccine in the media. Furthermore, differences of attitude between the Ministry of Health and the Prime Minister towards to the vaccine caused vaccination rates to stay low, even among HCW. Due to the number of anti-vaccine campaigns in the media and on the Internet, the acceptance of vaccination among HCW was significantly less than expected [20]. Accurate reporting by the media of the safety and efficacy of influenza vaccines and the importance of vaccines for the public health would likely have a positive influence on vaccine uptake. Uncertain or negative reporting about the vaccine is detrimental to vaccination efforts. In vaccination campaigns, governments should use the media effectively to provide safety. We think that our study may contribute in vaccination campaigns.

## Competing interests

The authors declare that they have no competing interests.

## Authors' contributions

DT participated in drafted the manuscript, participated in its design and coordination, ES conceived of the study, and participated in its design and coordination and helped to draft the manuscript. All authors read and approved the final manuscript.

## Pre-publication history

The pre-publication history for this paper can be accessed here:

http://www.biomedcentral.com/1471-2334/10/281/prepub
